# Book Notices

**Published:** 1883-02-20

**Authors:** 


### *BOOK NOTICES.*

RHEUMATISM, GOUT AND SOME ALLIED DISORDERS, by Morris Longstreet, M. D., one of the Attending Physicians of the Pennsylvania Hospital; Lecturer on Pathological Anatomy at the Jefferson Medical College, Philadelphia, Pa. New York: Wm. Wood & Co., oc. 280 pages.

A very interesting and instructive work treating exhaustively the subjects of gout and rheumatism, thier history, causes, pathology, course and symptoms, condition of skin, the urine, nervous complications, diagnosis and prognoses and treatment. Every practitioner would do well to procure and study this work.

POCKET THERAPEUTICS AND DOSE BOOK.—This is a most useful little work, contains Doses, Weights and Measures, Abbreviations, Writing Prescriptions, Index of Diseases, Poison Antidotes, Incompatibles, and everything of the kind needed by the practitioner. Two hundred and forty-eight pages, pocket size ; an epitome of nearly everything. Price, $1.00: G. D. Stewart & Co., Detroit, Michigan.

FOURTH ANNUAL REPORT STATE BOARD OF HEALTH OF ILLINOIS, 1882, contains an abstract of the proceedings for 1881, and various regular and quarterly meetings of the Board, with various interesting papers, in relation to medical colleges, small pox, etc., etc. Gotten up with evident care, neatness and ability. Sent us by Dr. John H. Rauch, Secretary.

PHYSICIANS' CLINICAL RECORD FOR HOSPITAL AND PRIVATE PRACTICE, with Memoranda for Examining Patients, Temperature, Charts, etc. Philadelphia: D. S. Brinton, 1881.

A well arranged blank book wherein to make notes of cases under the heads of date, pulse, respiration, temperature; other symptoms, treatment and remarks.

